# Robotic assisted anterior lumbar interbody fusion with the use of indocyanine green and posterior spinal fusion

**DOI:** 10.3171/2025.4.FOCVID2521

**Published:** 2025-07-01

**Authors:** Isabella Decker, Evelyn Hunter, Lauren Jiyeon Seo, Anna Landen, C. Rory Goodwin, Melissa Erickson, Sabino Zani, Muhammad M. Abd-El-Barr

**Affiliations:** 1 Department of Neurosurgery, Duke Health, Durham, North Carolina;; 2 Department of Orthopaedic Surgery, Duke Health, Durham, North Carolina; and; 3 Department of Surgery, Duke Health, Durham, North Carolina

**Keywords:** robotic surgery, R-ALIF, robotic ALIF, da Vinci robot, percutaneous pedicle screw fixation

## Abstract

A 50-year-old male with grade I spondylolisthesis at L5–S1 was treated surgically with a robotic anterior lumbar interbody fusion (R-ALIF). A general surgeon used the da Vinci robotic system to access the disc space and indocyanine green to visualize the vessels including the aortic bifurcation and middle sacral artery. A neurosurgeon completed the discectomy and fusion through a suprapubic GelPOINT Mini. R-ALIF combined with robotic percutaneous pedicle screw fixation resulted in no significant complications, minimal blood loss, a 4-hour surgical time, reduced patient-reported pain, and adequate cage placement.

The video can be found here: https://stream.cadmore.media/r10.3171/2025.4.FOCVID2521

## Transcript

### 0:27 Anterior Lumbar Interbody Fusion.

Anterior lumbar interbody fusion (ALIF) is a well-established surgical intervention for addressing a spectrum of degenerative pathologies affecting the lumbar spine, including degenerative disc disease (DDD), disc herniation, radiculopathy, and spondylolisthesis. Traditionally, ALIF has been performed using open surgical approaches, which, while effective, is associated with significant morbidity, including increased blood loss and potential injury to surrounding structures. Similarly, there has been an exponential growth in the interest in the use of robotics for posterior spinal procedures, including the placement of lumbosacral pedicle screws. There is increasing evidence that robotic-assisted placement of pedicle screws is associated with increased accuracy compared to other modalities, such as freehand, fluoroscopy-guided, or even navigation systems. Recent advancements in robotic surgical systems have facilitated a paradigm shift in spinal procedures. The integration of robotic technology into anterior spinal fusions allows for enhanced precision in accessing the retroperitoneal space^1^ while minimizing intraoperative blood loss^2–5^ and mitigating iatrogenic damage to adjacent tissues.^1–4,6^ This minimally invasive approach has demonstrated potential benefits in reducing perioperative complications,^1–3^ expediting postoperative recovery,^2,6^ and improving overall patient outcomes.^1,5^ Studies are warranted to elucidate the long-term advantages of robotic-assisted ALIF in comparison to conventional techniques.

### 1:56 Clinical Presentation.

A 50-year-old male presented with symptoms of back and radiating leg pain that were refractory to conservative management, including physical therapy, neuropathic pain modulators, NSAIDs, and acetaminophen. Imaging was significant for a grade 1 spondylolisthesis at L5–S1.

Diagnostic workup included a lumbar MRI, x-ray, and SPECT-CT, which showed greatest uptake in the L5–S1 region. Due to the findings of the spondylolisthesis at L5–S1 and the uptake around the disc space, the patient underwent bilateral transforaminal injection of lidocaine and dexamethasone at L5–S1. This injection provided temporary relief of the patient’s symptoms. Patient was scheduled for a robotic-assisted anterior lumbar interbody fusion (ALIF) with posterior spinal fusion at the L5–S1 level. The robotic-assisted approach was selected to reduce visceral manipulation, tissue damage, and the risk for abdominal hernias.

### 2:50 Patient Consent.

Written informed consent was obtained from the patient for the robotic procedure, as well as for the collection and sharing of his medical data and procedural videos for research and publication purposes. All identifying information was anonymized to maintain confidentiality.

### 3:08 Surgical Procedure.

Following induction of general anesthesia and the administration of perioperative antibiotics and tranexamic acid, the patient underwent endotracheal intubation. An indwelling Foley catheter was placed. Neuromonitoring leads were placed for intraoperative nerve monitoring. The patient was positioned on a Trumpf surgical bed in the supine position with arms in the "Superman" position, as demonstrated in the photo. Anterior access to the retroperitoneal space was gained using the da Vinci robotic system. Five incisions were made for port placement: 1) a 5-mm left upper quadrant incision for the optical trocar, using a 0° 5-mm scope, 2) two 8-mm robotic ports placed in the left and right lower quadrants, 3) two additional 8-mm robotic ports in the left and right midabdomen for instrument arms, 4) an 8-mm suprapubic port for manual access and future placement of a GelPort. After docking the da Vinci arms, the patient was placed in the Trendelenburg position at 27° to gain anterior access to the lumbar spine. This Trendelenburg position allowed for the disc space to be perpendicular to the floor, allowing for shorter working distance and improved surgical ergonomics compared to the traditional approach. Pneumoperitoneum was developed to 15 mm Hg. The abdominal contents were displaced superiorly or laterally, providing a clear operative field. Upon retraction of the peritoneal sac and retroperitoneal structures, the posterior abdominal wall was exposed, revealing the bifurcation of the abdominal aorta into the common iliac arteries crossing the L5–S1 intervertebral disc space. Ureters were visualized laterally and protected throughout the procedure, and the presacral nerves were mobilized. Indocyanine green (ICG) is used as a near-infrared fluorescent dye to visualize blood flow in real time. After intravenous injection, ICG binds to plasma proteins, then circulates through the vasculature. When exposed to near-infrared light, it emits fluorescence, allowing for clear delineation of arteries and vascular structures. ICG fluorescence imaging was performed intraoperatively to visualize the bifurcation of the common iliac arteries as well as the middle sacral artery to reduce risk of vascular injury. The middle sacral artery was ligated with 2-0 silk and transected to prevent excessive bleeding. The anterior longitudinal ligament (ALL) was then revealed via dissection of retroperitoneal fat and incised to access the disc space. After visualization of the intervertebral disc space, a 3-cm silk suture was used as a measuring guide to determine the width of the disc space for optimal selection of the interbody spacer and cage. A 25-gauge needle was placed in the L5–S1 disc space to confirm placement, and then the area was marked with cautery.

### 5:55 Disc Excision.

The patient was moved out of Trendelenburg position to supine. The suprapubic port was exchanged for a GelPOINT Mini to facilitate instrument exchange while maintaining pneumoperitoneum during the disc excision. A discectomy was performed using a sheathed scalpel and disc material was removed using a pituitary rongeur. A Cobb retractor was utilized to further clear the bony endplate and prepare the disc space for interbody cage placement in usual fashion.

### 6:19 Cage Placement.

Following preparation of the disc space, an expandable trial was placed to further open the disc space and to assess initial fit. After confirming appropriate sizing, the spacer was replaced with the Hedron IA metal interbody cage (Globus Medical) prepacked with osteogenic bone graft (Cerapedics). Using a modified inserter, two shims were malleted into L5. This modified inserter is enclosed—allowing for safer placement of the shims. Intraoperative lateral and AP x-ray imaging confirmed proper placement of the interbody cage. After shim placement, locking mechanism was engaged. During neuromonitoring, a transient loss of right lower extremity motor signals was noted but resolved intraoperatively, with no evidence of malpositioning. Hemostasis was ensured with Floseal application, and the robotic system was detached. A general surgeon performed superficial and dermal closure without deep layer approximation.

### 7:30 Percutaneous Pedicle Screw Fixation.

A staged posterior fixation was performed 4 days after the ALIF procedure, giving time to ensure adequate indirect decompression, using the ExcelsiusGPS robotic system (Globus Medical) to enhance stability, prevent cage subsidence, and promote fusion. Percutaneous screws were placed bilaterally, followed by two rods. The L5–S1 joint was exposed and decorticated. Both autograft and allograft were placed to constitute a supplemental posterolateral fusion. Placement of screws was accurate in postoperative CT.

### 8:00 Outcomes and Future Directions.

The patient experienced no significant complications, with full restoration of motor function upon awakening. Estimated blood loss was minimal on both surgeries, and the total operative time for the ALIF was 4 hours. The patient felt that his preoperative back and leg pain had improved, so no formal decompression was deemed necessary. Postoperative imaging confirmed midsagittal cage placement with adequate angulation. The patient was advanced to a regular diet and was discharged on postoperative pedicle screw placement day 2 with standard follow-up scheduled. Although the operative time for the robotic ALIF is longer than the open approach, the novelty of the technique requires practice and familiarization to reduce operative time. Robotic ALIF may be the safest approach for several patients, including severely obese patients and those with extensive abdominal surgery. Further exploration into the clinical uses of the da Vinci robot will reduce operative time, make surgery more accessible, and improve outcomes for patients.

**8:58 References.^1–8^**

## Disclosures

Mrs. Landen reported personal fees from Medtronic outside the submitted work. Dr. Goodwin reported grants from the Robert Wood Johnson Foundation Harold Amos Medical Faculty Development Program, the Food and Drug Administration, NIH (1R01DE031053-01A1), Stryker, Duke Bass Connections, and Carlsmed; personal fees from Medtronic and DePuy; serving as editor for *Spine*; and a patent application outside the current work. Dr. Erickson reported grants from Globus and Medtronic and personal fees from DePuy outside the submitted work. Dr. Zani reported consulting for Intuitive, Medtronic, and Asensus outside the submitted work. Dr. Abd-El-Barr reported consulting for Globus Medical, TrackX, BrainLab, and Arthrex outside the submitted work.

## Author Contributions

Primary surgeon: Abd-El-Barr. Assistant surgeon: Seo, Erickson, Zani. Editing and drafting the video and abstract: Decker, Hunter, Seo, Goodwin, Abd-El-Barr. Critically revising the work: Decker, Hunter, Seo, Goodwin, Abd-El-Barr. Reviewed submitted version of the work: Decker, Hunter, Seo, Goodwin, Erickson, Zani, Abd-El-Barr. Approved the final version of the work on behalf of all authors: Decker. Supervision: Decker, Zani, Abd-El-Barr. Physician assistant: Landen.

## Supplemental Information

### Patient Informed Consent

The necessary patient informed consent was obtained in this study.
